# A risk score system based on a six-microRNA signature predicts the overall survival of patients with ovarian cancer

**DOI:** 10.1186/s13048-022-00980-8

**Published:** 2022-05-06

**Authors:** Min Zhou, Tao Wu, Yuan Yuan, Shu-Juan Dong, Zhi-Ming Zhang, Yan Wang, Jing Wang

**Affiliations:** 1 Department of Gynecologic Cancer, Shaanxi Provincial Cancer Hospital, No. 309 Yanta West Road, Shaanxi 710061 Xi’an, People’s Republic of China; 2 Department of Obstetrics and Gynecology, Shaanxi Provincial Rehabilitation Hospital, Xi’an, Shaanxi People’s Republic of China; 3 Department of Clinical Laboratory, Xi’an Central Hospital, Xi’an, Shaanxi People’s Republic of China; 4 Department of Gynecology, Xi’an Central Hospital, No.161 five West Road, Xi’an, Shaanxi People’s Republic of China

**Keywords:** Ovarian cancer, microRNA signature, Prognosis, Bioinformatics analysis, TCGA

## Abstract

**Background:**

Ovarian cancer (OVC) is a devastating disease worldwide; therefore the identification of prognostic biomarkers is urgently needed. We aimed to determine a robust microRNA signature-based risk score system that could predict the overall survival (OS) of patients with OVC.

**Methods:**

We extracted the microRNA expression profiles and corresponding clinical data of 467 OVC patients from The Cancer Genome Atlas (TCGA) database and further divided this data into training, validation and complete cohorts. The key prognostic microRNAs for OVC were identified and evaluated by robust likelihood-based survival analysis (RLSA) and multivariable Cox regression. Time-dependent receiver operating characteristic (ROC) curves were then constructed to evaluate the prognostic performance of these microRNAs. A total of 172 ovarian cancer samples and 162 normal ovarian tissues were used to verify the credibility and accuracy of the selected markers of the TCGA cohort by quantitative real-time polymerase chain reaction (PCR).

**Results:**

We successfully established a risk score system based on a six-microRNA signature (hsa-miR-3074-5p, hsa-miR-758-3p, hsa-miR-877-5p, hsa-miR-760, hsa-miR-342-5p, and hsa-miR-6509-5p). This microRNA based system is able to characterize patients as either high or low risk. The OS of OVC patients, with either high or low risk, was significantly different when compared in the training cohort (*p* < 0.001), the validation cohort (*p* < 0.001) and the complete cohort (*p* < 0.001). Analysis of clinical samples further demonstrated that these microRNAs were aberrantly expressed in OVC tissues. The six-miRNA-based signature was correlated with the prognosis of OVC patients (*p* < 0.001).

**Conclusions:**

The study established a novel risk score system that is predictive of patient prognosis and is a potentially useful guide for the personalized treatment of OVC patients.

**Supplementary Information:**

The online version contains supplementary material available at 10.1186/s13048-022-00980-8.

## Background

Ovarian cancer (OVC) is a highly aggressive gynaecological malignancy. It is characterized by early onset of dissemination and high recurrence rate. As a consequence, the majority of patients have advanced disease at diagnosis [1]. The responses rate are expected in over 80% of women who receive standard platinum and paclitaxel-based treatment after upfront surgery. Overall, five-year survival rates range between 44% for all stages combined. However, the prognosis of patients with advanced stage disease is still dismal, with a 5-year survival ranging from 15 to 25% [2, 3]. Therefore, a refinement of the current clinic-pathologic risk assessment is needed to guide therapeutic strategies and to improve prognosis, using additional predictive biomarkers.

MicroRNAs regulate many biological function, including cell growth, differentiation and apoptosis. Because of their existence in almost all body fluids, microRNAs are thought of as new non-invasive biomarkers [4]. Studies have reported that miRNAs play a crucial role in the occurrence and development of OVC [5, 6]. In addition, several studies have demonstrated that miRNAs could be used as independent prognostic markers of patients with OVC [7, 8]. However, the prognostic value of a single microRNA is limited. It would be possible to identify a integrated signature with higher accuracy by combining multiple factors and proposing prognostic criteria [9, 10].

Recently, bioinformatics tools facilitates the identification of more potential prognostic biomarkers by mining data about mRNA, microRNA and long non-coding RNA. Lu et al. constructed a seven-miRNA-based prognostic signature for predicting the OS of patients with head and neck squamous cell carcinoma [11]. Wei et al. established a model based on nine-immune-related long non-coding RNAs using integrated bioinformatics analysis, which exhibited credible predictive power for the prognosis of pancreatic cancer [12]. Several signatures based on multiple microRNAs have been reported to predict the prognosis of OVC [13, 14]. However, these studies were based on a limited number of patients, or were only applicable for certain specific subgroups. Therefore, there is a need to develop a more effective and robust prognostic signature to provide guidance for the management of patients.

In this study, we comprehensively analyzed The Cancer Genome Atlas (TCGA) database to develop a new prognostic microRNA signature, and then established a novel risk score system that was capable of predicting the overall survival (OS) for OVC patients. In addition, we use clinical samples verify the prognostic value of the miRNA-based signature. The risk score system that is based on multiple microRNA signatures might provide novel insights into prognostic stratification and individualized management of OVC patients.

## Materials and methods

### Data processing

**Fig. 1:**
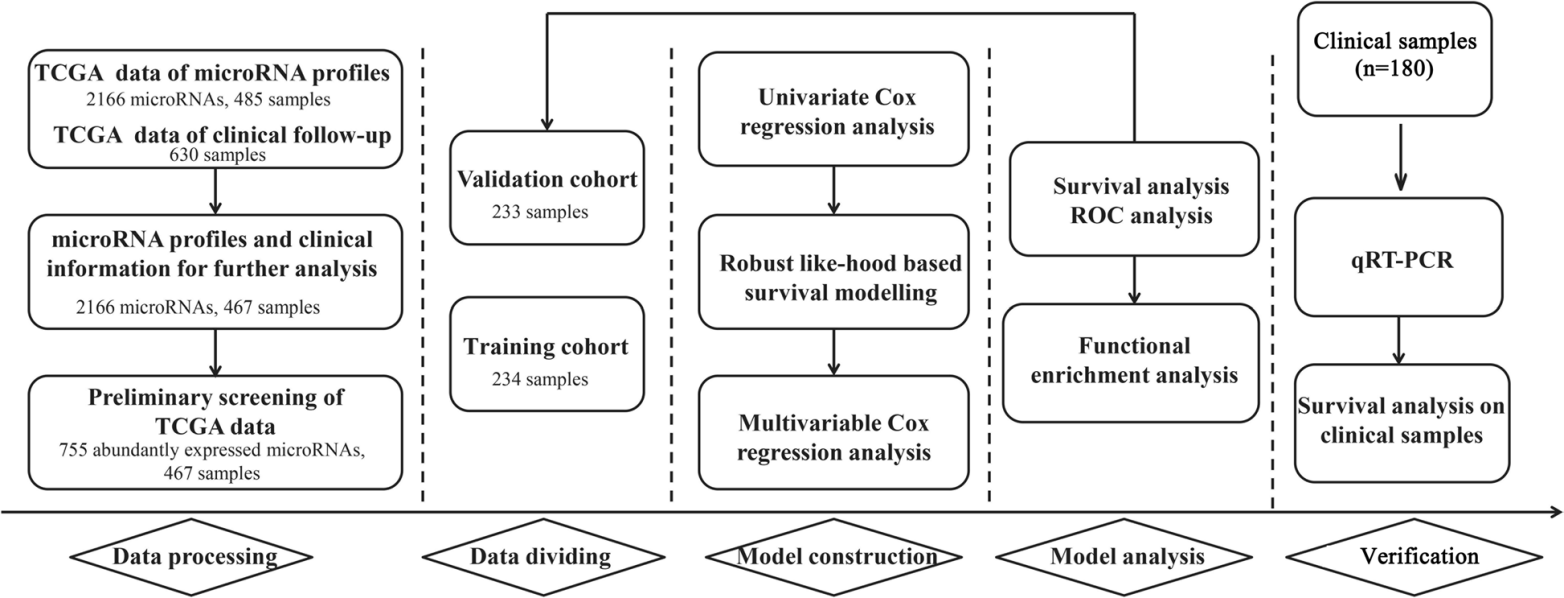
Flow chart showing the procedures involved in this study

Level 3 OVC microRNA sequencing (microRNA-seq) data and corresponding level 1 clinical data relating to TCGA-OVC were downloaded by the UCSC Xena browser (https://xenabrowser.net/). Figure 1 shows a flowchart for the study procedure. Our study included 2166 microRNA expression profiles from samples acquired from 485 patients and clinical follow-up data of 630 patients. Combining these two datasets by intersection, we obtained 2166 microRNA expression profiles from the samples of 467 patients. 755 of the microRNAs were abundantly expressed by obtaining the microRNA expression profiles. We defined a microRNA as being abundantly expressed when it matched two criteria: 1) expression level was above 0; and 2) the microRNA appeared in more than 50% of all specimens.

### The identification of microRNAs that are related to prognosis

We identified microRNAs that were significantly associated with OS by using univariate cox proportional hazard (CoxPH) regression analysis [15]. In order to identify a robust panel of microRNAs with the best prognostic ability and the minimum number of microRNAs, we performed a robust likelihood-based survival analysis (RSLA) [16]. A panel of microRNAs were selected by using the Akaike information criterion (AIC) value as a cut-off value. For convenience, *N* is used to refer the sample size of the training cohort. Then, the algorithm of the RSLA is summarized as follows:

1. Divide the training cohort again into a sub-training cohort with 2 *N*/3 samples and a sub-validation cohort with *N*/3 samples randomly. And then calculate the likelihood value, loglik*, which is a goodness-of-fit, for each microRNA.
2. Repeat the above step 10 times. In other words, the cross-validation is repeated 10 times independently. Thus, 10 loglik* values are yielded for each microRNA. Then calculate the meanloglik* value for each microRNA. The microRNA with the largest mean loglik* value is selected as the best microRNA, *g* (1).
3. Each two-microRNA model always including the best microRNA,*g* (1) is tested to find out the next best microRNA, *g* (2).
4. Continue the above steps, we can obtain a series of *K* models M_1_ = *g* (1), M_2_ = *g* (1) + *g* (2), …, M_K-1_ = *g* (1) + *g* (2) + … + *g*(K-1), M_K_ = *g* (1) + *g* (2) + … + *g*(*K*)..
5. Compute the AIC value for each model, and the model with smallest AIC is selected as an optimal model. The AIC value is calculated as -2loglik* + 2 *K*.

### Establishment of a risk score system

All selected prognosis-related microRNAs were included in multivariable Cox regression analysis. A risk score formula was subsequently established using the expression values and the coefficients of multivariable Cox regression analysis [14], as shown in Eq. 1.

1 $$Risk\ score=\sum \beta i\times Mi$$

In Eq. 1, Mi refers to the expression level of a microRNA, while βi refers to the coefficient obtained from multivariate Cox regression analysis. The median risk score was regarded as the cut-off value, thus OVC patients could be assigned to either a high-risk or low-risk group. Time-dependent receiver operating characteristic (ROC) analyses were then applied to test the 5-year and 10-year survival rate. Area under the curve (AUC) values were then calculated to estimate the prediction ability of the signature.

### Functional enrichment analysis

The target genes for the prognostic microRNAs were derived for further analysis using miRWalk 3 (http://mirwalk.umm.uni-heidelberg.de/). A gene targeted by half or more than half of the prognosis-related microRNAs was defined as an ‘overlapping target gene’. Kyoto Encyclopedia of Genes and Genomes (KEGG) pathway enrichment analysis was conducted on the overlapping target genes using ClusterProfiler software with a *P* value< 0.01 as the cut-off criterion [17]. We then used Cytoscape software 3.5.1. to create a network that featured significantly enriched KEGG pathways and related genes. Gene Ontology (GO) enrichment analysis was performed to reveal the significant enriched biological process, molecular function and cellular component.

### Clinical samples

A total of 172 frozen ovarian cancer samples and 162 normal ovarian tissues were collected between January 2013 and December 2016 from the Department of Gynecology Cancer at the Shaanxi Provincial Tumor Hospital (Xi’an, China). These tissues were immediately snap-frozen in liquid nitrogen and stored at-80 °C until the isolation of RNA. The clinic-pathological data including age, International Federation of Gynecology and Obstetrics (FIGO) stage, histological type, grade, lymph node metastasis, surgical debulking and chemotherapy response were retrospectively reviewed from patient electrical medical records. None of these patients had received radiotherapy or chemotherapy prior to surgery. Detailed patient characteristics are summarized in Supplementary Table 1. All enrolled patients were followed up until April 20th, 2020, or death. The median follow-up were 38 months (IQR,22–76 months). Overall survival was defined from the date of diagnosis to death or the end of follow-up (survivor); This study was approved by the Biomedical Ethics Committee of Xi’an Jiaotong University Health Science Centre (No.: 2019–672). (Xi’an, China), and written informed consent was obtained from all the patients.

### qRT-PCR

Total RNA was extracted fromtissues using Trizol reagent (Invitrogen, Carlsbad, CA, USA) in accordance with the manufacturer’sprotocol. cDNAs were synthesized using a PrimeScriptRT reagent Kit (Takara, Japan). Then, qRT-PCR was performed usingtheKAKA SYBR FAST qPCR Kit (Kapa Biosystems, USA) and a 7900HT Fast Real-Time PCR System (Applied Biosystems, Foster City, CA, USA). The small nuclear RNA, U6,was used as an internal control. The primer sequences were synthesized by Shanghai GenePharma Co., Ltd. In detail, the primer sequences are summarized in Supplementary Table 2.The 2^−∆∆Ct^ method was used to analyze expression levels that were normalized to the endogenous internal control.

### Statistical analysis

Cox regression analysis (univariate or multivariable), robust likelihood-based survival analysis and ROC analysis were all performed in R software by using the Survival package, the Rbsurv package and the Survival ROC package, respectively. Differences in survival between the two groups were also analyzed in R software using the Kaplan-Meier method and a log-rank test. Groups were compared by one-way analysis of variance (ANOVA) or the Student’s t-test. Pearson’s chi-squared test, or Fisher’s exact test, was used to evaluate categorical variables. Statistical significance was set at *P*-value < 0.05.

## Results

### Classification of TCGA data

All 467 cases were randomly divided into a training cohort (*n* = 234, used to identify key microRNAs) and a validation cohort (*n* = 233, used to validate the microRNA signature). There were no significant differences between these two cohorts regarding age, clinical stage, histological grade, lymphatic invasion, living status, or residual tumor status (Table 1).

**Table 1:** Clinical characteristics of the two cohorts of ovarian cancer patients from TCGA datasets

Parameters	Discovery cohort (***n*** = 234)	Validation cohort (***n*** = 233)	***p***-value	Method
Age (Mean ± SD)	59.6 ± 11.5	60.2 ± 11.7	0.57	t-test
Clinical stage				
I/II	14	16	0.82	χ2 test
III/IV	219	214		
Null	1	3		
Histologic grade				
G1	1	0	0.63	Fisher’s exact test
G2	29	32		
G3	201	190		
Null	3	11		
Lymphatic invasion				
No	28	32	0.67	χ2 test
Yes	54	51		
Null	152	150		
Living status				
Living	87	88	0.97	χ2 test
Dead	147	145		
Tumor residual disease				
1-10 mm	96	112	0.51	χ2 test
11-20 mm	14	15		
> 20 mm	43	37		
Null	81	69		

### Identification of microRNAs related to prognosis

Univariable cox regression analysis of the training cohort identified 59 statistically significant microRNAs (*P* < 0.05) (Supplementary Table 3). The six prognosis-related microRNAs (hsa-miR-3074-5p, hsa-miR-758-3p, hsa-miR-877-5p, hsa-miR-760, hsa-miR-342-5p and hsa-miR-6509-5p) were picked out using Robust likelihood-based survival analysis (Table 2).

**Table 2:** A prognosis-related microRNA signature in the discovery cohort

MicroRNA	Gene ID	nloglik	AIC	Selected
hsa-miR-3074-5p	5	666.12	1334.24	*
hsa-miR-758-3p	4	663.16	1330.33	*
hsa-miR-877-5p	16	660.98	1327.96	*
hsa-miR-760	10	658.13	1324.26	*
hsa-miR-342-5p	14	655.4	1320.8	*
hsa-miR-6509-5p	20	654.24	1320.49	*
hsa-miR-410-3p	11	654.08	1322.16	
hsa-miR-654-3p	15	654.06	1324.11	
hsa-miR-4473	8	652.13	1322.25	
hsa-miR-551a	3	651.35	1322.7	

### Developing a risk score based on a 6-microRNA signature

we performed multivariable Cox proportional regression analysis based on the six prognosis-related microRNAs determined by the training cohort. The consequent risk score was established as follows:

$$Risk\ score=\left(-0.05171\ast hsa- miR-3074-5p\right)+\left(0.22493\ast hsa- miR-758-3p\right)+\left(-0.06977\ast hsa- miR-877-5p\right)+\left(-0.16781\ast hsa- miR-760\right)+\left(-0.24161\ast hsa- miR-342-5p\right)+\left(-0.12669\ast hsa- miR-6509-5p\right)$$

**Fig. 2:**
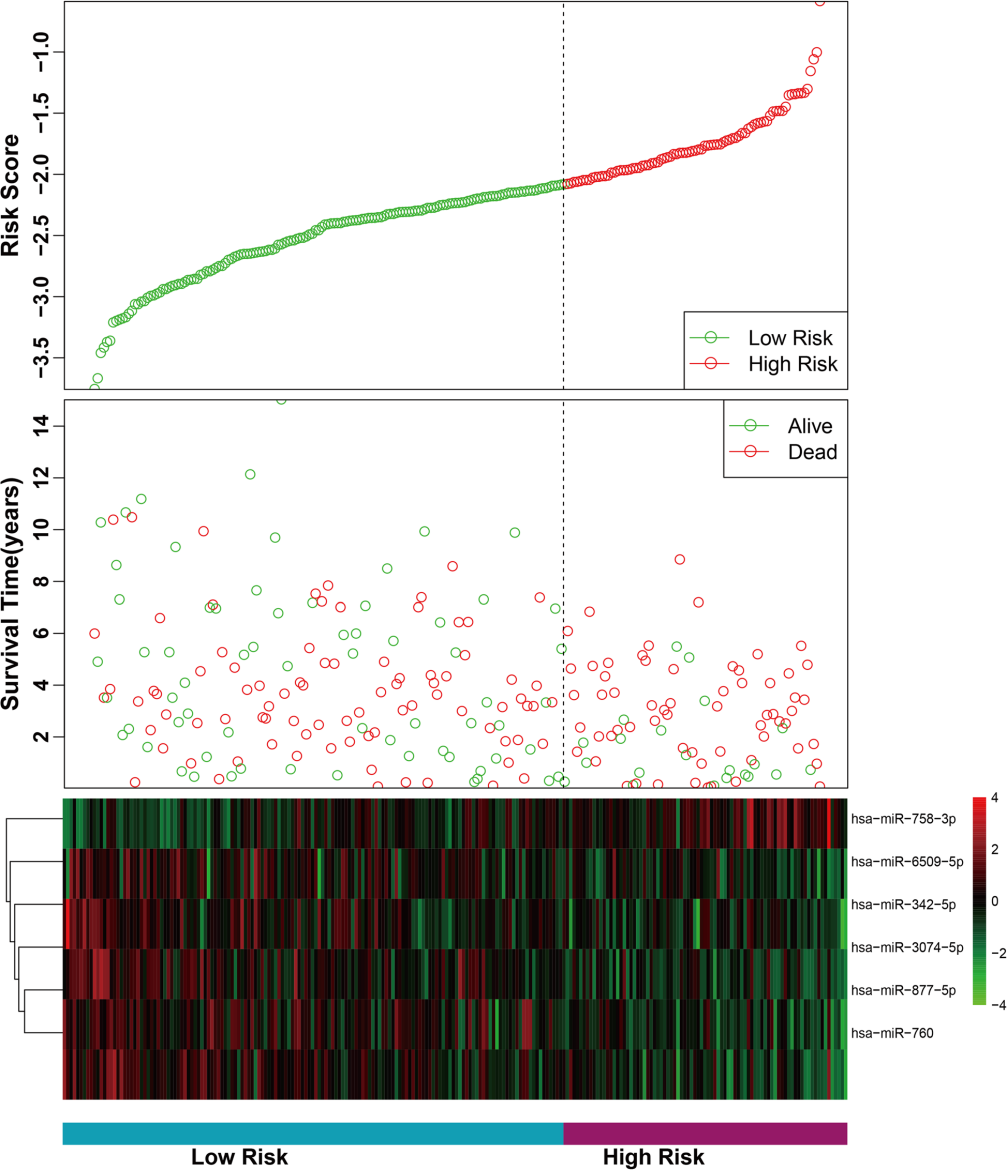
MicroRNA risk score analysis for the training cohort. From top to bottom: risk score distribution, distribution of patient survival status and a heat map of the six microRNAs for the two groups

Patients were assigned to high-risk group (*n* = 117) and low-risk group (*n* = 117) according to the Youden index (− 2.083) of risk score. As shown in Fig. 2, the survival status and risk score distribution analyses further demonstrated high risk group had more death cases than low risk group. Furthermore, the expression levels of hsa-miR-758-3p tended to be higher among patients in the high risk group; the opposite trend was shown for the other 5 microRNAs. A Pearson correlation coefficient was calculated as −0.32 with *P*-value < 10^−6^. Thus, the survival time for OVC patients was negatively correlated with their risk scores.

### Performance and validation of the prognostic risk score system

**Fig. 3:**
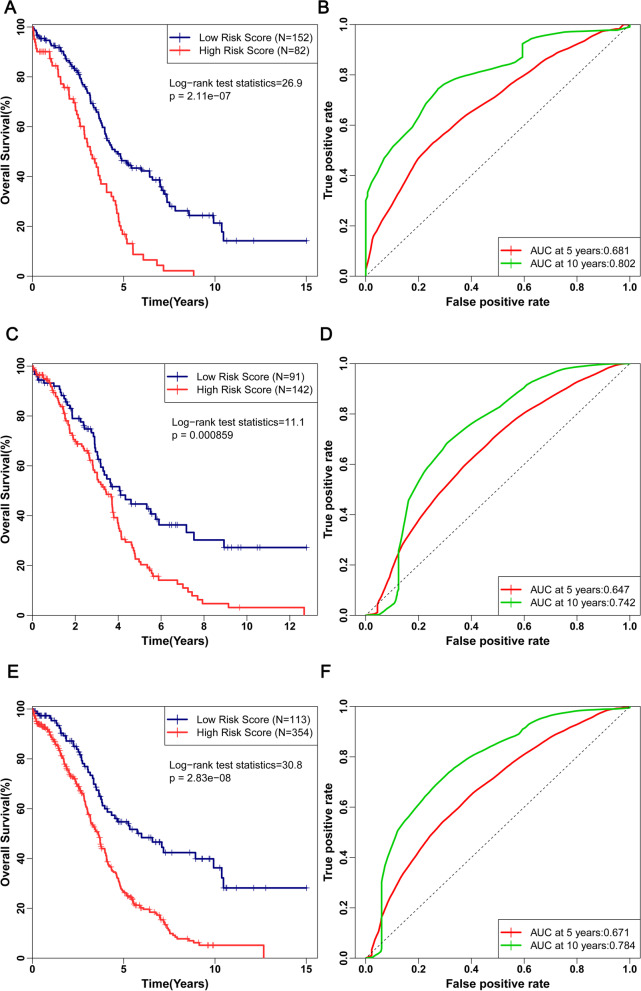
Kaplan-Meier curves for the low risk and high risk groups of patients of the training cohort (**A**), validation cohort (**C**) and complete cohort (**E**). The ROC curves for predicting OS for patients with OVC in the training cohort (**B**), validation cohort (**D**) and complete cohort (**F**) in accordance with the risk score

As shown in Fig. 3A, OS was significantly higher in patients with low risk score group compared with patients with high risk score (*P* < 0.0001). The prognosis-related microRNA signature possess a remarkable ability to predict survival with AUC values of 0.681 and 0.802 for 5-year and 10-year OS, respectively by time-dependent ROC analysis (Fig. 3B). To reinforce the prediction ability of the prognostic risk score system, we also performed survival analysis for the validation cohort and the complete cohort. In accordance with the results of the training cohort, the survival curves (Fig. 3C and E) indicated a highly significant difference between the low risk and high risk groups (all *P* < 0.001). In terms of the time dependent ROC analysis for the validation cohort, the AUC was 0.647 and 0.742 for 5-year and 10-year OS, respectively (Fig. 3D). In addition, the AUC value was 0.671 and 0.784 for the 5-year and 10-year OS for the complete cohort, respectively (Fig. 3F). As most patients involved in the present TCGA data had clinical stages of III or IV. Thus, We conducted subgroup analyses for late-stage ovarian cancer. As shown in Supplementary Fig. 1. K-M survival curve suggested that the OS of patients (stage III, stage IV and stage III-IV) with high-risk group was significantly lower than that in the patients(stage III, stage IV and stage III-IV) with low-risk group (all *P* < 0.001). It was also found that the proposed risk score had an outstanding ability in stratifying OS of patients with clinical stages of III or IV in terms of 10 years survival.

### Functional characteristics of the genes targeted by the prognosis-related microRNAs

**Fig. 4:**
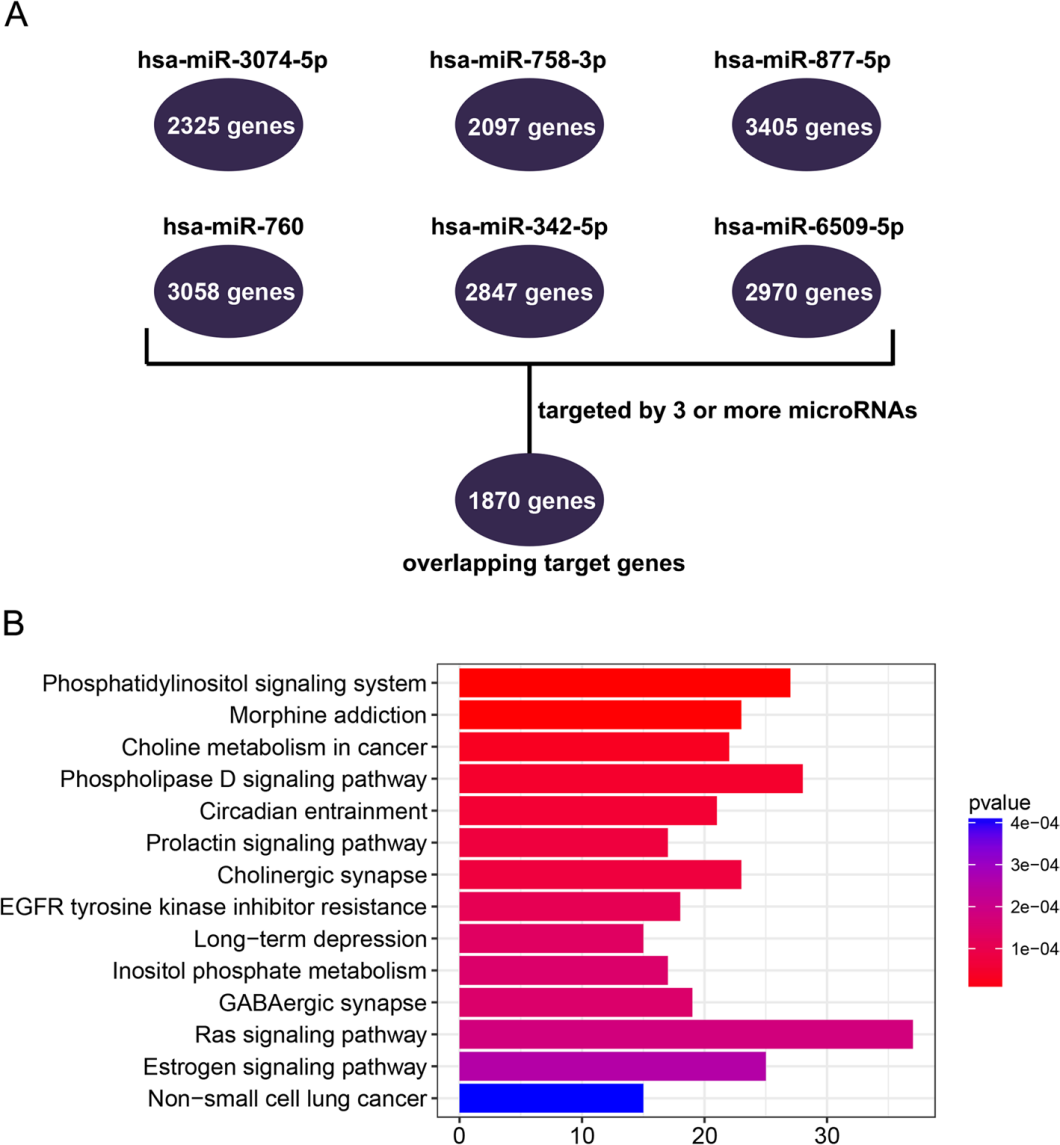
**A** Overlapping target genes and **B** significantly enriched KEGG pathways

Using the six microRNAs in miRWalk 3, we derived six cohorts of target genes belonging to each microRNA. When duplicate genes were removed, there were 2325, 2097, 2405, 3058, 2847, and 2970 target genes found for hsa-miR-3074-5p, hsa-miR-758-3p, hsa-miR-877-5p, hsa-miR-760, hsa-miR-342-5p, and hsa-miR-6509-5p, respectively. A gene targeted by 3 or more microRNAs (and therefore appeared in at least 3 cohorts) was defined as an ‘overlapping target gene’. In total, we identified 1870 overlapping target genes (Fig. 4A). To explore the molecular underlying mechanisms of the overlapping target genes, we conducted KEGG functional enrichment analysis, in which pathways with *P* < 0.01 were defined as significantly enriched pathways (Supplementary Table 4). The results showed that these genes were predominantly involved in pathways including phosphatidylinositol signaling system, morphine addiction, choline metabolism in cancer, the phospholipase D signaling pathway, circadian entrainment, the prolactin signaling pathway, cholinergic synapses and EGFR tyrosine kinase inhibitor resistance (Fig. 4B). According to the GO enrichment analysis, intracellular signal transduction, nervous system development and phospholipid biosynthetic process were the most significantly enriched biological process; protein binding, transcription factor activity, sequence-specific DNA binding and metal ion binding were the most significantly enriched molecular function. The top 15 GO terms were shown in Supplementary Table 5.

### Network of significantly enriched KEGG pathways and related genes

**Fig. 5:**
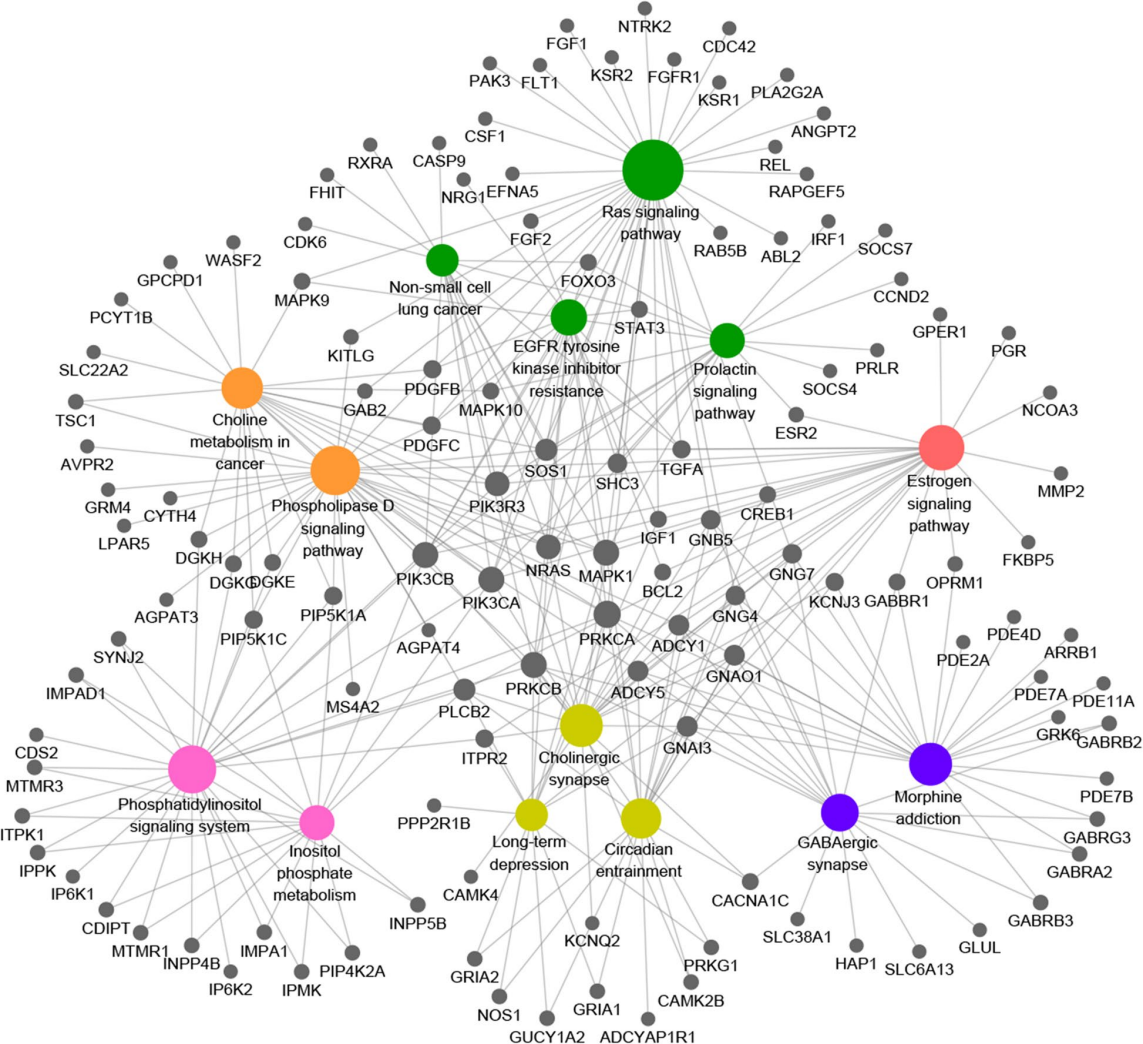
The coloured circles represent significantly enriched KEGG pathways, while the dark circles represent related genes. KEGG pathways are shown in the same colour if they are involved in similar functions. The size of the node reflects the degree of connectively of each node

Figure 5 shows a network that includes significantly enriched KEGG pathways and related genes as visualized by Cytoscape. These results suggested that PRKCA, MAPK1, PRKCB, PIK3CB, PIK3CA, NRAS, PIK3R3, SOS1 and PLCB2 were involved in at least half of the KEGG pathways. Therefore, these genes may participate in regulating the progression of OVC.

### Verification of the selected markers using clinical tissues

**Fig. 6:**
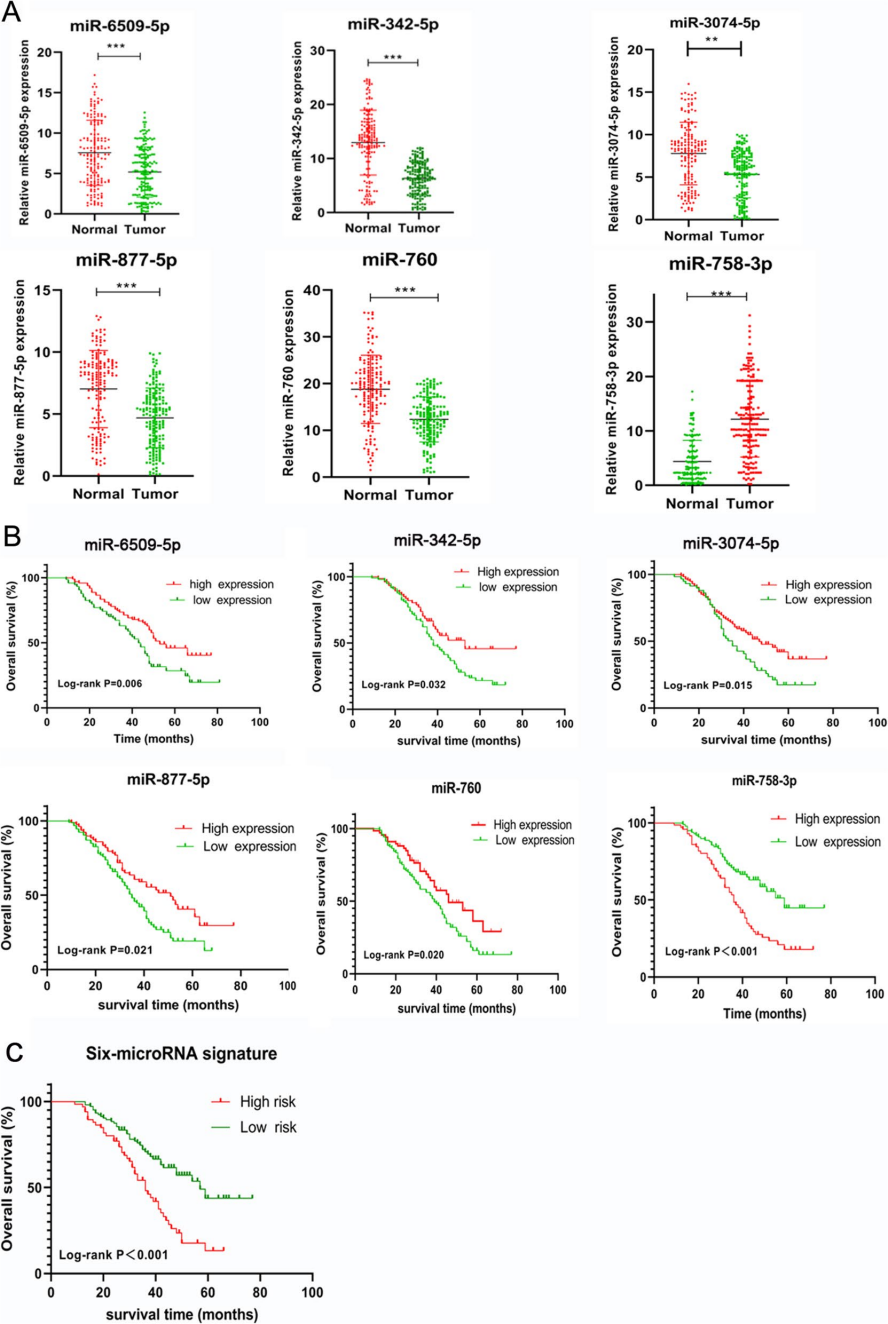
**A** The expression levels of the six microRNAs in 172 OVC samples and 162 normal ovaries by qRT-PCR. **, *p* < 0.01,***, *p* < 0.001. **B** Overall survival in OVC patients with high or low expression index of the six different microRNAs. **C** Overall survival in OVC patients with high risk score or low risk score

To investigate the expression of the selected microRNAs in OVC, we assessed the expression levels of the 6 microRNAs in 172 OVC samples and 162 normal ovaries by qRT-PCR. As shown in Fig. 6A, the results indicated that the expression of miR-6509-5p, miR-342-5p, miR-3074-5p, miR-877-5p, and miR-760, were dramatically reduced in OVC tissues, compared with normal tissues (all *P* < 0.05). However, miR-758-3p expression was markedly increased in OVC simples compared with normal ovaries (*P* < 0.05). To investigate the prognostic significance of the selected microRNAs, we performed Kaplan-Meier survival analysis. We selected the median value of the micro-RNAs from the entire dataset as the cutoff point. These results suggested that median survival of patients with higher levels of miR-6509-5p (53 months), miR-342-5p (53 months), miR-3074-5p (47 months), miR-877-5p (51 months), and miR-760 (46 months) had better survival than those with lower levels of miR-6509-5p (43 months), miR-342-5p (38 months), miR-3074-5p(35 monhs), miR-877-5p(35 months), and miR-760 (39 months) (all *p*-value < 0.05, Fig. 6B). Patients who over-expressed miR-758-3p had a shorter median survival time (36 months) than its low expression (59 months, *p*-value < 0.05). The recruited cohort were divided into two groups: low risk score (*n* = 105) and high risk score (*n* = 67) using the six-microRNA signature. The relationships between different microRNA expression levels, six-miRNA-based signature and clinicopathological parameters are summarized in Supplementary Table 6. Patients in high risk group were associated with advanced TNM stage (*P* = 0.019), lymph node metastasis (*P* = 0.032) and chemo- resistant (*P* = 0.022). The median OS for patients with high risk score group was 37 months and low risk group was 57 months. As seen in Fig. 6C, OS was significantly higher in patients with low risk groups compared with high risk groups (*p*-value < 0.01).

## Discussion

The prognostic role of microRNAs in OVC has been extensively investigated [13, 18]. However, a single microRNA cannot be used as an accurate prognostic marker with high sensitivity and specificity. In a recent paper, Korsunsky et al. analyzed the co-expression and regulatory structure of microRNAs from 20 patients with advanced OVC in order to construct a regulatory signature for predicting prognosis [13]. However, this signature was limited by the small sample size and was restricted to cases of advanced OVC. Others research studies were also associated with the same problems [19, 20]. In the current study, we identified a six-microRNAs based risk model that can predict the prognosis of patients with OVC. The algorithm employed for robust likelihood-based survival analysis ensured the best combination of microRNAs. Consequently, the present study could provide a more effective and robust diagnostic signature in comparison with those derived from previous studies.

According to our ROC analysis, the combined microRNA signature, which includes hsa-miR-3074-5p, hsa-miR-758-3p, hsa-miR-877-5p, hsa-miR-760, hsa-miR-342-5p, and hsa-miR-6509-5p, showed a pronounced ability to predict the clinical outcome of OVC patients. Hence, the risk score derived in this study, based upon a specific microRNA signature, can act as a promising index for clinical prognosis. Furthermore, we conducted functional enrichment analysis to identify the underlying molecular mechanisms associated with microRNA signature and OVC. Our clinical experiments also proved that the microRNAs used in our signature represented biomarkers for the prognosis of OVC. Our findings may, therefore, provide novel insights for elucidating the pathogenesis and prognostic prediction of OVC.

We observed that higher hsa-miR-758-3p levels acted as a predictor for a worse OS among the 6 microRNAs; however, the other microRNAs played opposing prognostic roles in patients with OVC. Our clinical experiments reconfirmed the findings from our bioinformatics analysis. Hsa-miR-760 has been shown to be closely related to the proliferation or metastasis of cancer cells in many cancers by regulating certain target genes [21]. In another study, Xiong et al. demonstrated the up-regulation of hsa-miR-877-5p in hepatocellular carcinoma [22]. Other work has shown that hsa-miR-877 is able to suppress the DME expression associated with APAP-induced hepatotoxicity and contributes to an adaptive response in hepatocytes [23]. However, the precise function of hsa-miR-877-5p in OVC remains unclear. Some studies have reported that hsa-miR-342-5p is associated with poor survival of patients with sarcoidosis and basal-like breast cancer [24, 25]. However, none of the existing studies have investigated the prognositic ability of hsa-miR-342-5p in OVC. Previous work has demonstrated the involvement of hsa-miR-3074-5p in embryo implantation, the aberrant expression of which has been associated with recurrent miscarriage [26]. In the current study, it seems rational to speculate that hsa-miR-3074-5p may play a prognostic role in OVC. Further studies are now required to determine whether this speculation can be fully validated. At present, there is only a limited literature database relating to hsa-miR-758-3p; further research will significantly enhance our understanding of how hsa-miR-758-3p may be associated with the functional mechanisms of OVC.

We investigated significantly enriched KEGG pathways and the key target genes associated with these pathways. Our findings were in line with a range of previous studies, with respect to choline metabolism [27], the Ras-Raf-ERK signaling pathway [28], the estrogen receptor pathway [29], MAPK1 [30], PRKCB [31], PIK3CA [31], NRAS [32], PIK3R3 [33] and SOS1 [34]. These pathways have all been demonstrated to play vital roles in the aggressiveness, growth, and metastasis of OVC. Further biological experiments are needed to reveal the underlying molecular mechanisms associated with the present microRNA signature.

The limitations of this study are as follows. First, although the 6 microRNAs have been validated by the recruited cohort to be correlated with the prognosis of OVC patients, further validations should be carried out to test the performance of the 6-microRNA risk score system in clinical patients management including the prognosis and therapy. Second, we only had limited TCGA clinical data for our patients; thus, we could not perform subgroup analysis by stratifying more clinicopathological factors. Third, the precise molecular mechanisms underlying the action of the six-microRNAs remains to be further explored in patients with OVC. The proposed key pathways regulated by miRNA through modulation of the gene-expression should be validated by experimentation at the functional level using a model system in the future.

## Conclusions

In summary, we constructed a prognostic model for OVC based on a six-microRNA signature (hsa-miR-3074-5p, hsa-miR-758-3p, hsa-miR-877-5p, hsa-miR-760, hsa-miR-342-5p and hsa-miR-6509-5p). The risk score system exhibited significant prognostic value for OVC patients. Clinical experiments further proved that the six-microRNA signature were useful prognostic biomarkers for OVC and may provide novel insights for unraveling the pathogenesis of OVC. These six microRNAs could potentially serve as biomarkers for prognostic stratification and individualized surveillance of patients with OVC.

## Supplementary Information

**Additional file 1: Supplementary Table 1.** Characteristics of the patients at baseline.

**Additional file 2: Supplementary Table 2.** Primers used for quantitative RT-PCR.

**Additional file 3: Supplementary Table 3.** Statistically significant microRNA obtained by univariable cox regression analysis in the discovery cohort.

**Additional file 4: Supplementary Table 4.** Significantly enriched KEGG pathways based on overlapping target genes.

**Additional file 5: Supplementary Table 5.** GO enrichment analysis of the target genes.

**Additional file 6: Supplementary Table 6.** Association of six miRNAs and clinical features.

**Additional file 7: Supplementary Fig. 1.** Performance and validation of the prognostic risk score system of subgroup of late stage patients. Kaplan-Meier curves for the low risk and high risk groups of patients with stage III (A), patients with stage IV(C) and patients with stage III and stage IV(E). The ROC curves for predicting OS for patients with stage III (B), patients with stage IV (D) and patients with stage III and stage IV(F) in accordance with the risk score.

## Data Availability

The dataset of clinical samples from the Shaanxi Provincial Tumor Hospital that was analysed during the current study is available from the corresponding author on reasonable request. The dataset of TCGA-OV is available from the following URL: https://xenabrowser.net/datapages/?cohort=TCGA%20Ovarian%20Cancer%20(OV)&removeHub=https%3A%2F%2Fxena.treehouse.gi.ucsc.edu%3A443.

## Abbreviations

OVC: Ovarian cancer
TCGA: The Cancer Genome Atlas
CoxPH: Cox proportional hazard
OS: Overall survival
qRT-PCR: Quantitative real-time PCR
ROC: Receiver operating characteristic
AUC: The area under the curve
KEGG: Kyoto Encyclopedia of Genes and Genomes.
