# Supplementary material for: A risk score system based on a six-microRNA signature predicts the overall survival of patients with ovarian cancer
Source: J Ovarian Res. 2022 May 6;15:54. doi: 10.1186/s13048-022-00980-8 (PMC9074233; doi:10.1186/s13048-022-00980-8)
Supplement: Supplementary file 1 — Additional file 1: Supplementary Table 1. Characteristics of the patients at baseline. [file 13048_2022_980_MOESM1_ESM.docx]

**Supplementary table 1:Characteristics of the patients at baseline.**

| Characteristics | Ovarian cancer, N (%) | Normal, N (%) |
| --- | --- | --- |
| Total, n (%) | 172 | 162 |
| Median age (Range) | 55 (29-75) | 55 (21-73) |
| Age (yr) |  |  |
| ≤55 | 76 | 102 |
| ＞55 | 96 | 60 |
| Histological type |  |  |
| Serous | 162 |  |
| Endometrioid | 10 |  |
| Histologic grade |  |  |
| G1/G2 | 11 |  |
| G3 | 161 |  |
| FIGO stage |  |  |
| I/II | 20 |  |
| III/IV | 152 |  |
| Lymph node metastasis |  |  |
| Positive | 92 |  |
| Negative | 54 |  |
| Not evaluable | 26 |  |
| Surgical debulking |  |  |
| Optimal | 110 |  |
| Sub-optimal | 62 |  |
| Chemotherapy |  |  |
| Chemoresistant | 36 |  |
| Chemosensitive | 136 |  |

Abbreviations: FIGO International Federation of Gynecology and Obstetrics

notes: we have removed 6 mucinous cases, 1 case with ovarian clear cell carcinoma and 1 case with ovarian small cell carcinoma in the revision.
