# Supplementary material for: A risk score system based on a six-microRNA signature predicts the overall survival of patients with ovarian cancer
Source: J Ovarian Res. 2022 May 6;15:54. doi: 10.1186/s13048-022-00980-8 (PMC9074233; doi:10.1186/s13048-022-00980-8)
Supplement: Supplementary file 2 — Additional file 2: Supplementary Table 2. Primers used for quantitative RT-PCR. [file 13048_2022_980_MOESM2_ESM.docx]

Supplementary Table 2. Primers used for quantitative RT-PCR.

| RNA | 5' to 3' |
| --- | --- |
| miR-3074-5p | Forward TTACACGGTTCTGCTGAACTG |
|  | Reverse TATCCTTCTTCACGACTCCTTCAC |
| miR-758-3p | Forward TCCTGTGCTTTGTGACCTGGT |
|  | Reverse GTGCAGGGTCCGAGGT |
| miR-877-5p | Forward GGCGAGTAGAGGAGATGGC |
|  | Reverse TATGGTTTGACGACTGTGTGAT |
| miR-760 | Forward CAATTCTAACGGCTCTGGGTC |
|  | Reverse TATGCTTGTTCTCGTCTCTGTGTC |
| miR-342-5P | Forward CTTGCTTGTATGAGGGGTGCTAT |
|  | Reverse TATGGTTGTTCACGAGTCCTTGTC |
| miR-6509-5P | Forward TCGGTCAGTGATTACGTAGTGGC |
|  | Reverse TATCCTTCTTCACGACTCCTTCAC |
| U6 | Forward CTCGCTTCGGCAGCACA |
|  | Reverse AACGCTTCACGAATTTGCGT |
