# Supplementary material for: A risk score system based on a six-microRNA signature predicts the overall survival of patients with ovarian cancer
Source: J Ovarian Res. 2022 May 6;15:54. doi: 10.1186/s13048-022-00980-8 (PMC9074233; doi:10.1186/s13048-022-00980-8)
Supplement: Supplementary file 3 — Additional file 3: Supplementary Table 3. Statistically significant microRNA obtained by univariable cox regression analysis in the discovery cohort. [file 13048_2022_980_MOESM3_ESM.docx]

Supplementary table 3: Statistically significant microRNA obtained by Univariable cox regression analysis in the discovery cohort.

| microRNA | HR | lowCI | highCI | p-value |
| --- | --- | --- | --- | --- |
| hsa-miR-377-5p | 1.23 | 1.09 | 1.39 | 0.00067 |
| hsa-miR-1301-3p | 0.75 | 0.63 | 0.9 | 0.0021 |
| hsa-miR-551a | 0.81 | 0.7 | 0.93 | 0.00322 |
| hsa-miR-758-3p | 1.19 | 1.06 | 1.34 | 0.00329 |
| hsa-miR-3074-5p | 0.83 | 0.73 | 0.94 | 0.00411 |
| hsa-miR-485-3p | 1.15 | 1.04 | 1.27 | 0.00536 |
| hsa-miR-605-5p | 1.31 | 1.08 | 1.59 | 0.00614 |
| hsa-miR-4473 | 0.68 | 0.51 | 0.9 | 0.00682 |
| hsa-miR-431-3p | 1.14 | 1.04 | 1.25 | 0.00683 |
| hsa-miR-760 | 0.84 | 0.74 | 0.95 | 0.00746 |
| hsa-miR-410-3p | 1.15 | 1.04 | 1.28 | 0.0077 |
| hsa-miR-654-5p | 1.18 | 1.04 | 1.33 | 0.00949 |
| hsa-miR-370-3p | 1.16 | 1.04 | 1.29 | 0.00999 |
| hsa-miR-342-5p | 0.79 | 0.65 | 0.94 | 0.01029 |
| hsa-miR-654-3p | 1.16 | 1.03 | 1.29 | 0.01159 |
| hsa-miR-877-5p | 0.83 | 0.71 | 0.96 | 0.01159 |
| hsa-miR-323a-5p | 1.26 | 1.05 | 1.5 | 0.01178 |
| hsa-miR-432-5p | 1.14 | 1.03 | 1.26 | 0.01215 |
| hsa-miR-20a-3p | 1.43 | 1.08 | 1.88 | 0.01242 |
| hsa-miR-6509-5p | 0.82 | 0.71 | 0.96 | 0.01247 |
| hsa-miR-187-3p | 0.9 | 0.82 | 0.98 | 0.01405 |
| hsa-miR-99b-5p | 0.74 | 0.59 | 0.94 | 0.0145 |
| hsa-miR-676-3p | 0.69 | 0.51 | 0.93 | 0.01515 |
| hsa-miR-485-5p | 1.18 | 1.03 | 1.36 | 0.01657 |
| hsa-miR-505-5p | 0.78 | 0.64 | 0.96 | 0.01795 |
| hsa-miR-29b-1-5p | 0.83 | 0.71 | 0.97 | 0.01885 |
| hsa-miR-2355-3p | 0.74 | 0.58 | 0.95 | 0.01928 |
| hsa-miR-887-3p | 0.83 | 0.71 | 0.97 | 0.02047 |
| hsa-miR-493-5p | 1.17 | 1.02 | 1.33 | 0.02063 |
| hsa-miR-6877-5p | 0.82 | 0.69 | 0.97 | 0.02284 |
| hsa-miR-487b-3p | 1.18 | 1.02 | 1.37 | 0.02323 |
| hsa-miR-127-3p | 1.14 | 1.02 | 1.27 | 0.02343 |
| hsa-miR-342-3p | 0.82 | 0.69 | 0.97 | 0.02367 |
| hsa-miR-1262 | 0.79 | 0.65 | 0.97 | 0.02514 |
| hsa-miR-23c | 0.77 | 0.61 | 0.97 | 0.02635 |
| hsa-miR-222-5p | 1.23 | 1.02 | 1.47 | 0.02682 |
| hsa-miR-765 | 0.75 | 0.59 | 0.97 | 0.02742 |
| hsa-miR-3187-3p | 0.85 | 0.74 | 0.98 | 0.02975 |
| hsa-miR-92a-3p | 1.26 | 1.02 | 1.55 | 0.03111 |
| hsa-miR-4728-3p | 1.18 | 1.01 | 1.37 | 0.03163 |
| hsa-miR-18a-5p | 1.27 | 1.02 | 1.58 | 0.03169 |
| hsa-miR-323a-3p | 1.13 | 1.01 | 1.26 | 0.03213 |
| hsa-miR-433-3p | 1.14 | 1.01 | 1.28 | 0.0339 |
| hsa-miR-589-3p | 0.81 | 0.66 | 0.98 | 0.03437 |
| hsa-miR-501-5p | 0.85 | 0.73 | 0.99 | 0.03455 |
| hsa-miR-370-5p | 1.15 | 1.01 | 1.31 | 0.03477 |
| hsa-miR-487a-5p | 1.14 | 1.01 | 1.28 | 0.03579 |
| hsa-miR-135b-5p | 0.85 | 0.73 | 0.99 | 0.03749 |
| hsa-miR-4664-5p | 0.78 | 0.62 | 0.99 | 0.04024 |
| hsa-miR-541-3p | 1.14 | 1.01 | 1.29 | 0.04048 |
| hsa-miR-191-5p | 0.82 | 0.68 | 0.99 | 0.04086 |
| hsa-miR-199a-5p | 1.13 | 1 | 1.27 | 0.04352 |
| hsa-miR-1284 | 0.66 | 0.44 | 0.99 | 0.04383 |
| hsa-miR-409-3p | 1.13 | 1 | 1.27 | 0.04499 |
| hsa-miR-501-3p | 0.87 | 0.76 | 1 | 0.04559 |
| hsa-miR-381-3p | 1.15 | 1 | 1.32 | 0.04788 |
| hsa-miR-337-3p | 1.15 | 1 | 1.32 | 0.04814 |
| hsa-miR-3614-5p | 0.89 | 0.79 | 1 | 0.0482 |
| hsa-miR-20b-5p | 0.89 | 0.79 | 1 | 0.04901 |
