# Supplementary material for: A risk score system based on a six-microRNA signature predicts the overall survival of patients with ovarian cancer
Source: J Ovarian Res. 2022 May 6;15:54. doi: 10.1186/s13048-022-00980-8 (PMC9074233; doi:10.1186/s13048-022-00980-8)
Supplement: Supplementary file 4 — Additional file 4: Supplementary Table 4. Significantly enriched KEGG pathways based on overlapping target genes. [file 13048_2022_980_MOESM4_ESM.docx]

**Supplementary table 4 :Significantly enriched KEGG pathways based on overlapping target genes.**

|  | ID | Description | GeneRatio | BgRatio | pvalue | p.adjust | qvalue | geneID | Count |
| --- | --- | --- | --- | --- | --- | --- | --- | --- | --- |
| hsa04070 | hsa04070 | Phosphatidylinositol signaling system | 27/647 | 99/7441 | 4.56E-08 | 1.39E-05 | 1.02E-05 | 10423/8760/8526/1608/160851/  3612/54928/8821/3633/9807/  51447/253430/64768/3705/3709/  8776/8897/5290/5291/8503/5305/  8394/23396/5330/5578/5579/  8871 | 27 |
| hsa05032 | hsa05032 | Morphine addiction | 23/647 | 91/7441 | 1.96E-06 | 0.000298 | 0.000219 | 107/111/408/2550/2555/  2561/2562/2567/2773/2775/  10681/2786/2788/2870/3760/  4988/50940/5138/5144/5150/  27115/5578/5579 | 23 |
| hsa05231 | hsa05231 | Choline metabolism in cancer | 22/647 | 99/7441 | 3.00E-05 | 0.003038 | 0.00223 | 8526/1608/160851/56261/5594/  5602/5601/4893/9468/5155/  56034/5290/5291/8503/8394/  23396/5578/5579/6582/6654/  7248/10163 | 22 |
| hsa04072 | hsa04072 | Phospholipase D signaling pathway | 28/647 | 146/7441 | 4.71E-05 | 0.003235 | 0.002375 | 107/111/56894/56895/554/  27128/8526/1608/160851/9846/  2914/4254/57121/5594/2206/  4893/5155/56034/5290/5291/  8503/8394/23396/5330/5578/  53358/6654/7248 | 28 |
| hsa04713 | hsa04713 | Circadian entrainment | 21/647 | 96/7441 | 5.79E-05 | 0.003235 | 0.002375 | 107/111/117/775/816/  1385/2773/2775/10681/2786/  2788/2890/2891/2977/3760/  5594/4842/5330/5578/5579/  5592 | 21 |
| hsa04917 | hsa04917 | Prolactin signaling pathway | 17/647 | 70/7441 | 7.36E-05 | 0.003235 | 0.002375 | 894/2100/2309/3659/5594/  5602/5601/4893/5290/5291/  8503/5618/53358/122809/30837/  6654/6774 | 17 |
| hsa04725 | hsa04725 | Cholinergic synapse | 23/647 | 112/7441 | 7.45E-05 | 0.003235 | 0.002375 | 107/111/596/775/816/  814/1385/2773/2775/10681/  2786/2788/3709/3760/3785/  5594/4893/5290/5291/8503/  5330/5578/5579 | 23 |
| hsa01521 | hsa01521 | EGFR tyrosine kinase inhibitor resistance | 18/647 | 79/7441 | 0.000111 | 0.004228 | 0.003104 | 596/2247/2309/3479/5594/  4893/3084/5155/56034/5290/  5291/8503/5578/5579/53358/  6654/6774/7039 | 18 |
| hsa04730 | hsa04730 | Long-term depression | 15/647 | 60/7441 | 0.000136 | 0.004295 | 0.003153 | 2773/2775/2890/2891/2977/  3479/3709/5594/4842/4893/  5330/5519/5578/5579/5592 | 15 |
| hsa00562 | hsa00562 | Inositol phosphate metabolism | 17/647 | 74/7441 | 0.000154 | 0.004295 | 0.003153 | 10423/3612/54928/8821/3633/  253430/64768/3705/8776/8897/  5290/5291/5305/8394/23396/  5330/8871 | 17 |
| hsa04727 | hsa04727 | GABAergic synapse | 19/647 | 88/7441 | 0.000155 | 0.004295 | 0.003153 | 107/111/775/2550/2555/  2561/2562/2567/2752/2773/  2775/10681/2786/2788/9001/  5578/5579/81539/6540 | 19 |
| hsa04014 | hsa04014 | Ras signaling pathway | 37/647 | 232/7441 | 0.000195 | 0.004931 | 0.00362 | 27/285/998/1435/1946/  2246/2247/2260/2321/9846/  10681/2786/2788/3479/4254/  8844/283455/5594/5602/5601/  4893/4915/5063/5155/56034/  5290/5291/8503/5320/5578/  5579/5869/9771/5966/53358/  6654/7039 | 37 |
| hsa04915 | hsa04915 | Estrogen signaling pathway | 25/647 | 137/7441 | 0.000269 | 0.006283 | 0.004612 | 107/111/596/1385/2100/  2289/2550/2773/2775/2852/  3709/3760/5594/4313/8202/  4893/4988/5241/5290/5291/  8503/5330/53358/6654/7039 | 25 |
| hsa05223 | hsa05223 | Non-small cell lung cancer | 15/647 | 66/7441 | 0.000421 | 0.009146 | 0.006714 | 842/1021/2272/2309/5594/  4893/5290/5291/8503/5578/  5579/6256/6654/6774/7039 | 15 |
