# Supplementary material for: A risk score system based on a six-microRNA signature predicts the overall survival of patients with ovarian cancer
Source: J Ovarian Res. 2022 May 6;15:54. doi: 10.1186/s13048-022-00980-8 (PMC9074233; doi:10.1186/s13048-022-00980-8)
Supplement: Supplementary file 5 — Additional file 5: Supplementary Table 5. GO enrichment analysis of the target genes. [file 13048_2022_980_MOESM5_ESM.docx]

**Supplementary table 5 : GO enrichment analysis of the target genes.**

| Category | Term | Count | P-Value |
| --- | --- | --- | --- |
| GOTERM_BP | intracellular signal transduction | 62 | 0.00023 |
| GOTERM_BP | nervous system development | 47 | 0.00037 |
| GOTERM_BP | phospholipid biosynthetic process | 12 | 0.001 |
| GOTERM_BP | transcription, DNA-templated | 227 | 0.0013 |
| GOTERM_BP | regulation of circadian rhythm | 13 | 0.0019 |
| GOTERM_BP | phosphatidylinositol biosynthetic process | 14 | 0.0029 |
| GOTERM_BP | histone deacetylation | 12 | 0.0035 |
| GOTERM_BP | negative regulation of transcription from RNA polymerase II promoter | 92 | 0.0038 |
| GOTERM_BP | positive regulation of transcription from RNA polymerase II promoter | 120 | 0.0042 |
| GOTERM_BP | regulation of protein heterodimerization activity | 5 | 0.0043 |
| GOTERM_BP | eye development | 9 | 0.0061 |
| GOTERM_BP | actin cytoskeleton organization | 23 | 0.0061 |
| GOTERM_BP | transforming growth factor beta receptor signaling pathway | 18 | 0.0062 |
| GOTERM_BP | protein transport | 54 | 0.0077 |
| GOTERM_BP | signal transduction | 137 | 0.0078 |
|  |  |  |  |
| GOTERM_CC | early endosome | 41 | 0.0001 |
| GOTERM_CC | cytosol | 370 | 0.0002 |
| GOTERM_CC | neuron projection | 41 | 0.00022 |
| GOTERM_CC | synaptic vesicle | 20 | 0.00086 |
| GOTERM_CC | intracellular | 159 | 0.0015 |
| GOTERM_CC | Golgi membrane | 79 | 0.0015 |
| GOTERM_CC | endosome | 36 | 0.0023 |
| GOTERM_CC | phagocytic vesicle | 11 | 0.0027 |
| GOTERM_CC | cytoplasm | 545 | 0.0028 |
| GOTERM_CC | endoplasmic reticulum membrane | 106 | 0.0039 |
| GOTERM_CC | lamellipodium | 27 | 0.0043 |
| GOTERM_CC | Golgi apparatus | 105 | 0.0056 |
| GOTERM_CC | endoplasmic reticulum | 101 | 0.0061 |
| GOTERM_CC | membrane | 240 | 0.0096 |
| GOTERM_CC | nucleus | 555 | 0.012 |
|  |  |  |  |
| GOTERM_MF | protein binding | 917 | 0.00016 |
| GOTERM_MF | transcription factor activity, sequence-specific DNA binding | 122 | 0.0012 |
| GOTERM_MF | metal ion binding | 235 | 0.0038 |
| GOTERM_MF | zinc ion binding | 140 | 0.0048 |
| GOTERM_MF | ubiquitin protein ligase activity | 30 | 0.007 |
| GOTERM_MF | protein serine/threonine kinase activity | 52 | 0.0078 |
| GOTERM_MF | SNAP receptor activity | 10 | 0.01 |
| GOTERM_MF | SNARE binding | 11 | 0.011 |
| GOTERM_MF | sequence-specific DNA binding | 67 | 0.012 |
| GOTERM_MF | myosin V binding | 6 | 0.019 |
| GOTERM_MF | vitamin D response element binding | 3 | 0.026 |
| GOTERM_MF | histone deacetylase activity | 9 | 0.03 |
| GOTERM_MF | 1-acylglycerol-3-phosphate O-acyltransferase activity | 6 | 0.031 |
| GOTERM_MF | ubiquitin-protein transferase activity | 43 | 0.036 |
| GOTERM_MF | chemorepellent activity | 7 | 0.04 |
| GOTERM_MF | DNA binding | 183 | 0.043 |
