# Supplementary material for: A risk score system based on a six-microRNA signature predicts the overall survival of patients with ovarian cancer
Source: J Ovarian Res. 2022 May 6;15:54. doi: 10.1186/s13048-022-00980-8 (PMC9074233; doi:10.1186/s13048-022-00980-8)
Supplement: Supplementary file 6 — Additional file 6: Supplementary Table 6. Association of six miRNAs and clinical features. [file 13048_2022_980_MOESM6_ESM.docx]

Supplementary Table 6. Association of six miRNAs and clinical features.

| Characteristics | N | miR-3074-5p | | P  value | miR-758-3p | | p  value | miR-877-5p | | p  value | miR-760 | | p  value | miR-342-5P | | p  value | miR-6509-5P | | p  value | six-microRNA signature | | p  value |
| --- | --- | --- | --- | --- | --- | --- | --- | --- | --- | --- | --- | --- | --- | --- | --- | --- | --- | --- | --- | --- | --- | --- |
|  |  | High | Low |  | High | Low |  | High | Low |  | High | Low |  | High | Low |  | High | Low |  | High risk | Low risk |  |
| Age (yr) |  |  |  | 0.759 |  |  | 0.162 |  |  | 0.142 |  |  | 0.302 |  |  | 0.890 |  |  | 0.195 |  |  | 0.900 |
| ≤55 | 76 | 38 | 48 |  | 33 | 43 |  | 35 | 41 |  | 32 | 44 |  | 41 | 34 |  | 40 | 36 |  | 30 | 46 |  |
| ＞55 | 96 | 40 | 46 |  | 52 | 44 |  | 55 | 41 |  | 48 | 48 |  | 52 | 45 |  | 41 | 55 |  | 37 | 59 |  |
| Histological type |  |  |  | 0.981 |  |  | 0.716 |  |  | 0.861 |  |  | 0.921 |  |  | 0.951 |  |  | 0.891 |  |  | 0.686 |
| Serous | 162 | 73 | 89 |  | 79 | 83 |  | 84 | 78 |  | 75 | 87 |  | 88 | 74 |  | 77 | 85 |  | 62 | 100 |  |
| Endometrioid | 10 | 5 | 5 |  | 6 | 4 |  | 6 | 4 |  | 5 | 5 |  | 5 | 5 |  | 4 | 6 |  | 5 | 5 |  |
| Histologic grade |  |  |  |  |  |  | 0.032 |  |  | 0.437 |  |  | 0.110 |  |  | 0.973 |  |  | 0.255 |  |  | 0.254 |
| G1/G2 | 11 | 4 | 7 | 0.759 | 2 | 9 |  | 7 | 4 |  | 8 | 3 |  | 6 | 5 |  | 7 | 4 |  | 2 | 9 |  |
| G3 | 161 | 74 | 87 |  | 83 | 78 |  | 83 | 78 |  | 77 | 84 |  | 87 | 74 |  | 74 | 87 |  | 65 | 96 |  |
| FIGO stage |  |  |  | 0.018 |  |  | 0.020 |  |  | 0.09 |  |  | 0.006 |  |  | 0.013 |  |  | 0.029 |  |  | 0.019 |
| I/II | 20 | 14 | 6 |  | 5 | 15 |  | 14 | 6 |  | 15 | 5 |  | 16 | 4 |  | 14 | 6 |  | 3 | 17 |  |
| III/IV | 152 | 64 | 88 |  | 80 | 72 |  | 76 | 76 |  | 65 | 87 |  | 77 | 75 |  | 67 | 85 |  | 64 | 88 |  |
| Lymph node metastasis |  |  |  | 0.043 |  |  | 0.016 |  |  | 0.003 |  |  | 0.106 |  |  | 0.023 |  |  | 0.124 |  |  | 0.032 |
| Positive | 92 | 50 | 42 |  | 58 | 34 |  | 38 | 54 |  | 45 | 47 |  | 40 | 52 |  | 39 | 53 |  | 42 | 50 |  |
| Negative | 54 | 20 | 34 |  | 23 | 31 |  | 36 | 18 |  | 19 | 35 |  | 34 | 20 |  | 30 | 24 |  | 15 | 39 |  |
| Not evaluable | 26 | 8 | 18 |  | 4 | 22 |  | 16 | 10 |  | 16 | 10 |  | 19 | 7 |  | 12 | 14 |  | 10 | 16 |  |
| Surgical debulking |  |  |  | 0.778 |  |  | 0.166 |  |  | 0.646 |  |  | 0.789 |  |  | 0.867 |  |  | 0.226 |  |  | 0.093 |
| Optimal | 110 | 49 | 61 |  | 50 | 60 |  | 59 | 51 |  | 52 | 58 |  | 60 | 50 |  | 48 | 62 |  | 48 | 62 |  |
| Suboptimal | 62 | 29 | 33 |  | 35 | 27 |  | 31 | 31 |  | 28 | 34 |  | 33 | 29 |  | 33 | 29 |  | 19 | 43 |  |
| chemotherapy |  |  |  | 0.017 |  |  | 0.019 |  |  | 0.149 |  |  | 0.779 |  |  | 0.015 |  |  | 0.025 |  |  | 0.022 |
| chemoresistant | 36 | 10 | 26 |  | 24 | 12 |  | 15 | 21 |  | 16 | 20 |  | 13 | 23 |  | 11 | 25 |  | 20 | 16 |  |
| chemosensitive | 136 | 68 | 68 |  | 61 | 75 |  | 75 | 61 |  | 64 | 72 |  | 80 | 56 |  | 70 | 66 |  | 47 | 89 |  |
