# Supplementary figures and images for: A risk score system based on a six-microRNA signature predicts the overall survival of patients with ovarian cancer
Source: J Ovarian Res. 2022 May 6;15:54. doi: 10.1186/s13048-022-00980-8 (PMC9074233; doi:10.1186/s13048-022-00980-8)

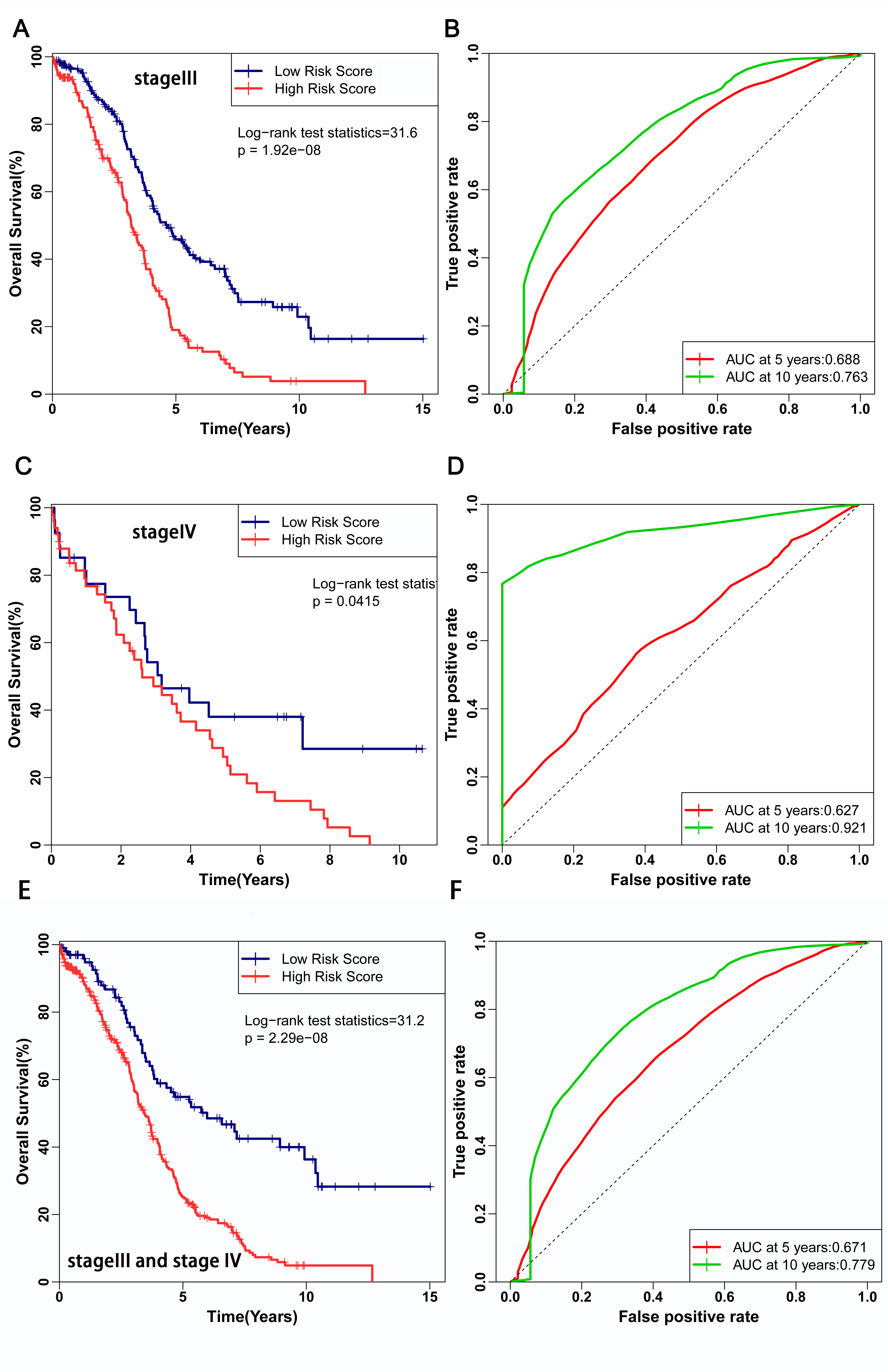

Supplement: Supplementary file 7 — Additional file 7: Supplementary Fig. 1. Performance and validation of the prognostic risk score system of subgroup of late stage patients. Kaplan-Meier curves for the low risk and high risk groups of patients with stage III (A), patients with stage IV(C) and patients with stage III and stage IV(E). The ROC curves for predicting OS for patients with stage III (B), patients with stage IV (D) and patients with stage III and stage IV(F) in accordance with the risk score. [file 13048_2022_980_MOESM7_ESM.tif]
